# Dynamics of Human Mitochondrial Complex I Assembly: Implications for Neurodegenerative Diseases

**DOI:** 10.3389/fmolb.2016.00043

**Published:** 2016-08-22

**Authors:** Gabriele Giachin, Romain Bouverot, Samira Acajjaoui, Serena Pantalone, Montserrat Soler-López

**Affiliations:** Structural Biology Group, European Synchrotron Radiation FacilityGrenoble, France

**Keywords:** complex I, MCIA, assembly factors, mitochondrial dysfunction, neurodegeneration, Alzheimer's disease, Parkinson's disease

## Abstract

Neurons are extremely energy demanding cells and highly dependent on the mitochondrial oxidative phosphorylation (OXPHOS) system. Mitochondria generate the energetic potential via the respiratory complexes I to IV, which constitute the electron transport chain (ETC), together with complex V. These redox reactions release energy in the form of ATP and also generate reactive oxygen species (ROS) that are involved in cell signaling but can eventually lead to oxidative stress. Complex I (CI or NADH:ubiquinone oxidoreductase) is the largest ETC enzyme, containing 44 subunits and the main contributor to ROS production. In recent years, the structure of the CI has become available and has provided new insights into CI assembly. A number of chaperones have been identified in the assembly and stability of the mature holo-CI, although they are not part of its final structure. Interestingly, CI dysfunction is the most common OXPHOS disorder in humans and defects in the CI assembly process are often observed. However, the dynamics of the events leading to CI biogenesis remain elusive, which precludes our understanding of how ETC malfunctioning affects neuronal integrity. Here, we review the current knowledge of the structural features of CI and its assembly factors and the potential role of CI misassembly in human disorders such as Complex I Deficiencies or Alzheimer's and Parkinson's diseases.

## Introduction

The major mechanisms mediating neuronal activity are all initially powered by the oxidative phosphorylation system (OXPHOS) in mitochondria, the so-called “powerhouse” of the cell (Hall et al., 2012). They generate energetic potential through the electron transport chain (ETC) which includes complex I (CI or NADH:ubiquinone oxidoreductase), complex II (CII or succinate:ubiquinone oxidoreductase), complex III (CIII or ubiquinol:cytochrome-*c* oxidoreductase), and complex IV (CIV or cytochrome-*c* oxidase). Together with complex V (CV or F_O_F_1_-ATP-synthase) they form what is usually called the OXPHOS system. Biogenesis of a functional OXPHOS system requires a large set (>92) of mitochondrial- and nuclear-encoded proteins (Koopman et al., 2013). Despite the critical importance of the CI in energy production, many aspects of its structure, assembly and activity are still poorly understood. Here, we provide an overview of the current knowledge of CI structure and the factors involved in its assembly, with an emphasis on the Mitochondrial Complex I Assembly (MCIA) complex. We then examine the supporting evidence that correlates CI dysfunction and misassembly with neurodegeneration, in particular Complex I Deficiencies, Alzheimer's and Parkinson's diseases. Overall, this review attempts to explore recent advances into the molecular mechanisms of CI assembly based on structural and clinical studies in order to provide a better understanding of its underlying mechanisms in neurodegenerative disorders.

## Mitochondrial energy production in neurons: The critical role of the oxidative phosphorylation system

As a first step in oxidative phosphorylation, fuel molecules (such as monosaccharides and fatty acids) are transferred to nicotinamide (NAD) and flavine adenine (FAD) nucleotides through glycolysis, Krebs cycle and β-oxidation and are then oxidized through the ETC. CI, CIII, and CIV generate proton force in the intermembrane space and their actions are facilitated by CII and electron transfer cofactors (i.e., ubiquinone and cytochrome-*c*). Proton translocation back to the mitochondrial matrix drives CV, which is coupled to ATP synthesis. Most of the ATP produced by CV is exchanged against cytosolic ADP through a specific adenine nucleotide carrier to supply the rest of the cell with energy and to maintain the ADP phosphorylation capacity of mitochondria (Lasserre et al., 2015).

Many inner membrane transporters are also driven by the electrochemical proton gradient, which is required for the maintenance of mitochondrial integrity and essential functions like apoptosis, innate immunity, redox control, calcium homeostasis, and several biosynthetic processes (Koopman et al., 2013).

The redox reactions involved in energy production generate reactive oxygen species (ROS), which have important roles in cell signaling and homeostasis. However, high levels of ROS can also lead to oxidative stress. ROS levels are particularly critical in the central nervous system (CNS) since neurons are extremely energy demanding but have limited glycolysis, making them highly dependent on an efficient OXPHOS. The ability of the brain to withstand oxidative stress is limited because of (a) high content of easily oxidizable substrates; (b) relatively low levels of antioxidants; (c) the endogenous generation of ROS *via* several specific reactions; (d) the elevated content of iron in specific areas of the human brain, and (e) CNS contains non-replicating neuronal cells which, once damaged, may be permanently dysfunctional or committed to apoptosis (Calabrese and Halpain, 2005; Cao and Fang, 2015; Wakatsuki et al., 2015). Thus, it is not surprising that mitochondria of synaptic origin can be highly affected in response to physiological or environmental alterations, with severe consequences for neuronal function and survival. Metabolically, there is evidence that both neurons and astrocytes rely on OXPHOS for ATP generation, whereas astrocytes also possess energy stores in the form of glycogen (Hertz et al., 2007; Belanger et al., 2011). Moreover, the ability of mitochondria to move within the cells is also critical in highly polarized cells like neurons. Data from rat brain mitochondria of non-synaptic origin have shown that ETC complex activities need to be reduced by at least 60% before major changes in ATP synthesis and oxygen consumption occur. However, in synaptic mitochondria, titration of various ETC complexes with specific inhibitors show that decreased CI, III, and IV activities of 25, 80, and 70%, respectively, result in major changes in rates of oxygen consumption and ATP synthesis (Telford et al., 2009). This suggests that in mitochondria of synaptic origin, CI activity has a major control of oxidative phosphorylation, such that when a relatively low threshold of 25% inhibition is exceeded, energy metabolism is compromised, and reduction in ATP synthesis ensues (Telford et al., 2009).

Even though the basic functional principles of most components of the ETC have been elucidated, the details are still being hotly debated. We now know that each complex in the chain functions with a unique mechanism and that there are no direct analogs with other enzymes (Sazanov, 2015).

## Mitochondrial respiratory complex I: Structure and function

**Figure 1:**
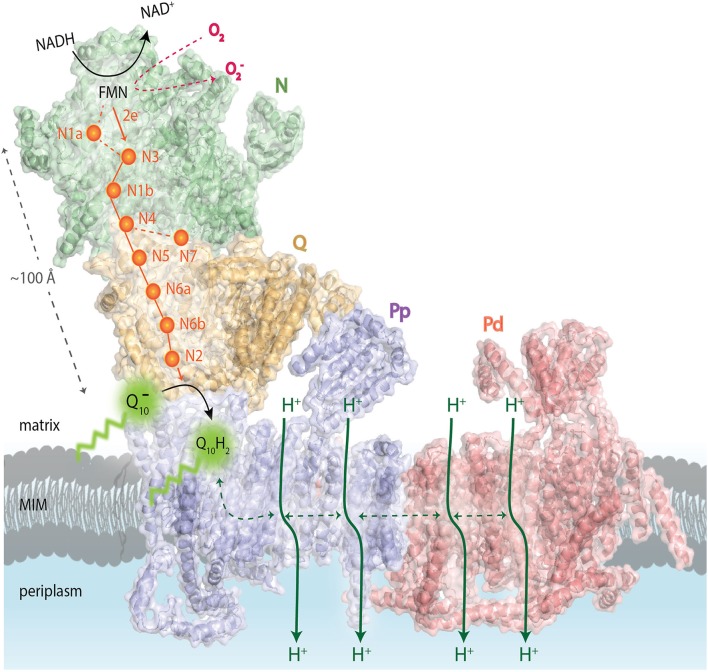
**Schematic overview of the functional complex I modules**. In the N-module (colored in green), the oxidation of NADH by a flavin mononucleotide (FMN) generates the release of two electrons (2e^−^) that enter into a chain of seven iron–sulfur (Fe-S) clusters (orange spheres). They are transferred from the terminal Fe-S cluster N2 onto a ubiquinone molecule (${\text{Q}}_{10}^{-}$) bound in the Q-module (in gold) which gets reduced (Q_10_H_2_). The reduction of ubiquinone induces conformational changes in the helices of the proton-translocating P-modules (Pp-proximal in violet and Pd-distal in salmon). As a result, a long chain of charged residues passing through the middle of the membrane connects to four putative pump sites consisting of separate proton input (from matrix) and output (to periplasm) channels. The reduced FMN cofactor also reacts with molecular oxygen to form reactive oxygen species, highlighted in red. MIM: mitochondrial inner membrane. Representation of mammalian mitochondrial CI model is based on the bovine heart cryo-EM structure Representation of mammalian mitochondrial CI model is based on the bovine heart cryo-EM structure (Vinothkumar et al., 2014; *PDB 4UQ8*).

CI is the largest and first enzyme of the ETC. It is essential for cellular energy production, providing about 40% of the proton motive force required for ATP synthesis. It oxidizes NADH to NAD^+^ and donates the released electrons to the electron carrier coenzyme Q_10_ (CoQ_10_, also known as ubiquinone), linking this process to the translocation of four protons from the mitochondrial matrix to the intermembrane space (Figure 1; Sazanov, 2014). These electron transfers generate superoxide (${\text{O}}_{2}^{\cdot -}$), which is the proximal mitochondrial ROS (St-Pierre et al., 2002; Kussmaul and Hirst, 2006; Murphy, 2009). Superoxide is normally converted to H_2_O_2_ by manganese superoxide dismutase (MnSOD); the latter can easily diffuse across the membranes and be quickly reduced to water by mitochondrial and cytoplasmic peroxiredoxins, catalases, and glutathione peroxidases (Cox et al., 2010; Li et al., 2013).

Mammalian CI is composed of 44 different subunits, all of which are integral components of the enzyme (approximately 1 MDa). Seven of these subunits (ND1, ND2, ND3, ND4, ND4L, ND5, and ND6) are encoded by the mitochondrial DNA (mtDNA) and the rest by the nuclear DNA (Vinothkumar et al., 2014). The prokaryotic counterpart of CI contains only the 14 highly conserved core subunits (a total mass of about 550 kDa), which harbor the bioenergetic functions. The extra counterparts seen in mammalian CI are thus called accessory or supernumerary subunits (Letts and Sazanov, 2015).

While the atomic-resolution structures and basic mechanisms of most respiratory complexes have been previously established, there are still large gaps in our understanding of the coupled electron transport and proton pumping in the OXPHOS complexes, being the CI the least understood (Letts and Sazanov, 2015). Crystal structures have been reported for prokaryotic CIs (also called NADH dehydrogenase-1) such as from *Escherichia coli* (Efremov and Sazanov, 2010, 2011), *T. thermophilus* (Baradaran et al., 2013), *P. denitrificans* (Sedlacek et al., 2014), and *A. aeolicus* (Yeh et al., 2000), reviewed in Berrisford and Sazanov (2009). Structural models are also available for eukaryotic CI, such as from the obligate aerobic yeast *Y. lipolytica* (Zickermann et al., 2015) and the fungus *Neurospora crassa* (Janssen et al., 2006). Furthermore, the recent cryo-EM structure of bovine heart CI has enabled the molecular modeling of the 14 core subunits plus 14 of the supernumerary subunits of the mammalian enzyme (Vinothkumar et al., 2014). Nevertheless, higher resolution data are still required for a full assignment of the rest of the supernumerary subunits to build a complete mammalian CI structural model (Letts and Sazanov, 2015).

Eukaryotic CI seems to be organized into four functional modules (Figure 1). It forms an L-shaped arm, composed of hydrophilic (matrix-protruding and peripheral) and lipophilic (inner membrane-embedded) segments (Clason et al., 2010). The two constituents have independent functions. The peripheral arm extends into the matrix and is responsible for the electron transfer reaction while the membrane arm catalyzes proton transport (Sazanov, 2015). The distal half of the matrix arm forms the N module that is made up of the central 75, 51, and 24-kDa subunits and contains the dehydrogenase site, formed by a flavin-mononucleotide (FMN) moiety and responsible for the oxidation of NADH to NAD^+^. The proximal half of the peripheral arm is the Q module. This reduces ubiquinone and is composed of the central 49-kDa, 30-kDa, PSST, and TYKY subunits, docking onto the membrane arm. A chain of eight iron-sulfur clusters runs over a distance of about 100 Å through the matrix arm allowing fast electron tunneling (Zickermann et al., 2015) in a very similar manner to that seen in prokaryotic CI, except that, owing to rotation of the 51- and 24-kDa subunits, the mammalian counterpart chains are more distal to the membrane and more divergent (Vinothkumar et al., 2014). The membrane arm contains 82 transmembrane helices (TMHs), 64 of them contributed by central subunits. The mammalian membrane domain is more strongly curved out of the membrane plane than the prokaryotic analog, mainly due to a transmembrane helix in subunit ND6 (Vinothkumar et al., 2014). The proximal pump module (P_P_) comprises the central subunits ND1, ND2, ND3, ND4L, and ND6, whereas the distal pump module (P_D_) contains the central subunits ND4 and ND5. The interior of the membrane arm is rich in polar and protonable residues (i.e., residues that may take up or release protons) constituting a remarkable hydrophilic central axis across all subunits (Zickermann et al., 2015).

Taken together, the overall architecture of the CI supports the idea that proton translocation is driven by long-range conformational changes. However, the energy-converting mechanism of CI remains largely unknown (Hirst and Roessler, 2015; Letts and Sazanov, 2015). Furthermore, many important structural details remain to be elucidated, including, for instance, the role of several single transmembrane ancillary subunits, which are proposed to form a scaffold structure holding the giant complex during its oscillatory movements; or when and how the different prosthetic groups are incorporated into the complex (Ghezzi and Zeviani, 2012). Along this line, CI has been long known to be inhibited by Zn^2+^, but the site of inhibitory Zn^2+^ binding remains to be identified (Hirst and Roessler, 2015).

## Complex I construction: The critical role of assembly factors

Specific pathways are required for the assembly of each ETC complex, including the transfer of nuclear and mtDNA-encoded ETC subunits into the inner membrane of mitochondria; the synthesis and incorporation of several prosthetic groups that form the catalytic redox cores of CI, CII, CIII, and CIV; and the ultimate formation of functionally active holo-complexes, which can also organize themselves in respiratory supercomplexes (Ghezzi and Zeviani, 2012). Additional systems verify the quality control of protein and non-protein components of the ETC complexes, contributing to the maintenance of their structural integrity, functional activity, and turnover. Thus, a highly regulated, extremely complex process is at work in mitochondria to control the formation, stability, interactions, function, and plasticity of the ETC (Ghezzi and Zeviani, 2012).

CI assembly is a complicated multistep process. Previous studies on mammalian CI biogenesis have used patient cells containing assembly defects to generate models of CI assembly (Lazarou et al., 2007). The presence of CI subunits in mobile (matrix-soluble) and/or immobile (membrane-bound) subassemblies *in vivo* indicates that a modular CI assembly pathway is operational in different mitochondrial compartments in the living cell (Dieteren et al., 2008). In addition, in human mitochondria CI is found exclusively as a component of respiratory supercomplexes, since it requires CIII for stability (Schagger et al., 2004; Moreno-Lastres et al., 2012). Although it is not known exactly how each subunit is assembled to form the mature CI in humans, a model for its assembly has been developed in recent years (Vogel et al., 2007b; Mckenzie and Ryan, 2010). Based on this, a stepwise assembly process of CI would involve pre-assembled modules of the peripheral matrix and membrane arms (Figure 1; Letts and Sazanov, 2015). Even if current data on CI assembly accounts for the addition of only 16 of the 30 mammalian mitochondria supernumerary subunits, they still provide strong clues concerning to which of the core subunits these supernumerary subunits are binding (Letts and Sazanov, 2015). Consistent with the highly hydrophobic nature of the proteins located in the P module, the membrane arm forms an assembly intermediate, including ND2, ND4, ND4L, ND5, and ND6 mitochondrial subunits before the progression into the mature holo-enzyme (Leman et al., 2015). Nevertheless, previous studies have demonstrated that subassemblies of nuclear DNA-encoded CI subunits could be formed in the absence of mtDNA-encoded subunits, suggesting that the presence of the mitochondrial-encoded subunits is not required for the formation of the peripheral arm subcomplex (Potluri et al., 2004). The Q module seems to assemble separately from the N module and associate with the membrane arm in a late stage intermediate of roughly 830 kDa. In a last step, the subunits of the tip of CI, i.e., subunits of the N module (core catalytic NDUFV1, NDUFV2, and NDUFS1 subunits and accessory NDUFV3, NDUFS4, 6 and NDUFA12 subunits) seem to be added to form a functional holo-enzyme (reviewed in Mckenzie and Ryan, 2010).

The integration of these subunits and insertion of cofactors into the nascent CI is aided by assembly factors, which tend to be specific for each complex but bind transiently without forming part of the final enzyme (Fernandez-Vizarra et al., 2009; Torraco et al., 2015; Sanchez-Caballero et al., 2016; Wirth et al., 2016). At least 13 assembly factors have now been identified and characterized to be involved in CI assembly (Table 1; Mimaki et al., 2012). They are encoded in the nucleus and are then delivered to the mitochondria. To ensure proper delivery, the majority contains a specific N-terminal signal pre-sequence, also called mitochondrial targeting sequence (MTS) that is removed after import by the mitochondrial processing peptidase. Nevertheless, some proteins do not contain a cleavable MTS. These include three CI assembly factors: TMEM126B, FOXRED1, and TIMMDC1 (Sanchez-Caballero et al., 2016). How assembly factors function is not known, but they may act as chaperones that stabilize the subcomplexes and help them to associate to other subcomplexes in order to build the complete enzyme (Vogel et al., 2007b; Andrews et al., 2013). The fact that crystal structures of the assembly factors are not available precludes our understanding of the mechanistic basis of these proteins at the molecular level (Guarani et al., 2014). These assembly chaperones may have additional functions besides their requirement for CI assembly, in line with the evidence that mitochondria are more than energy producers and are involved with various (sub)cellular processes that ultimately regulate mitochondrial metabolic activity (Vogel et al., 2007b).

**Table 1:** **CI assembly factors and interacting CI subunits**.

**Interactors**	**Description**	**CI interacting subunits**	**References**
ACAD9	Acyl-CoA dehydrogenase family member 9, mitochondrial	Core: NDUFS2, NDUFS3, NDUFS7, ND6	Nouws et al., 2010
		Accessory: NDUFA13, NDUFS5	
ECSIT	Evolutionarily conserved signaling intermediate in Toll pathway, mitochondrial	Core: NDUFS1, NDUFS2, NDUFS3, NDUFS8, ND1, ND4	Vogel et al., 2007a
		Accessory: NDUFA3, NDUFA8, NDUFA13, NDUFB1, NDUFB5, NDUFB8, NDUFB11, NDUFS5, NDUFC2	
FOXRED1	FAD-dependent oxidoreductase domain-containing protein 1	Accessory: NDUFS5	Formosa et al., 2015
NDUFAF1	Complex I intermediate-associated protein 30, mitochondrial	Core: NDUFS1, NDUFS3, NDUFS7, ND1	Vogel et al., 2005
		Accessory: NDUFA8, NDUFA9, NDUFA12, NDUFA13, NDUFB6, NDUFB11, NDUFS5	
NDUFAF2	NADH dehydrogenase [ubiquinone] 1 alpha subcomplex assembly factor 2	n.d.	Ogilvie et al., 2005
NDUFAF3	NADH dehydrogenase [ubiquinone] 1 alpha subcomplex assembly factor 3	Core: NDUFS2, NDUFS3, NDUFS7, ND6	Saada et al., 2009
		Accessory: NDUFA8, NDUFA13, NDUFB10, NDUFB11, NDUFS4, NDUFS5, NDUFS8	
NDUFAF4	NADH dehydrogenase [ubiquinone] 1 alpha subcomplex assembly factor 4	Core: NDUFS3, NDUFS7	Saada et al., 2008
		Accessory: NDUFA13, NDUFS5	
NDUFAF5	NADH dehydrogenase [ubiquinone] 1 alpha subcomplex assembly factor 5	Core: NDUFS3, NDUFS7	Sugiana et al., 2008
		Accessory: NDUFA10	
NDUFAF6	NADH dehydrogenase [ubiquinone] 1 alpha subcomplex assembly factor 6	n.d.	McKenzie et al., 2011
NDUFAF7	NADH dehydrogenase [ubiquinone] 1 alpha subcomplex assembly factor 7	Core: NDUFS7	Carilla-Latorre et al., 2010
		Accessory: NDUFA10	
NUBPL	Iron-sulfur protein NUBPL (Nucleotide-binding protein-like)	n.d.	Sheftel et al., 2009
TIMMDC1	Complex I assembly factor TIMMDC1 (Translocase of inner mitochondrial membrane domain-containing protein 1), mitochondrial	Core: NDUFS2, NDUFS7, NDUFS8, NDUFV1, ND1, ND2, ND4	Andrews et al., 2013
		Accessory: NDUFA3, NDUFA8, NDUFA9, NDUFA12, NDUFA13, NDUFB4, NDUFB5, NDUFB6, NDUFB8, NDUFB9, NDUFB10, NDUFS5, NDUFV3	
TMEM126B	Complex I assembly factor TMEM126B (Transmembrane protein 126B), mitochondrial	Accessory: NDUFA13	Heide et al., 2012

*Interactions curated by Intact (Orchard, 2014) and Biogrid (Chatraryamontri et al., 2015)*.

*n.d., no data available*.

### The mitochondrial complex I assembly (MCIA) complex

Most currently identified CI assembly factors are involved in early assembly and more specifically in the incorporation of the hydrophobic membrane subunits. The CI assembly factors NDUFAF1, ACAD9, ECSIT, TMEM126B, and TIMMDC1 form the MCIA complex (Heide et al., 2012), which was first identified in rat heart mitochondria and then in human osteosarcoma 143B cells by complexome profiling (Heide et al., 2012). The MCIA complex seems to associate with the 370-kDa subcomplex intermediate, consisting of subunits of the membrane P-proximal submodule (Figure 1; Lazarou et al., 2007; Andrews et al., 2013).

#### NDUFAF1

**Figure 2:**
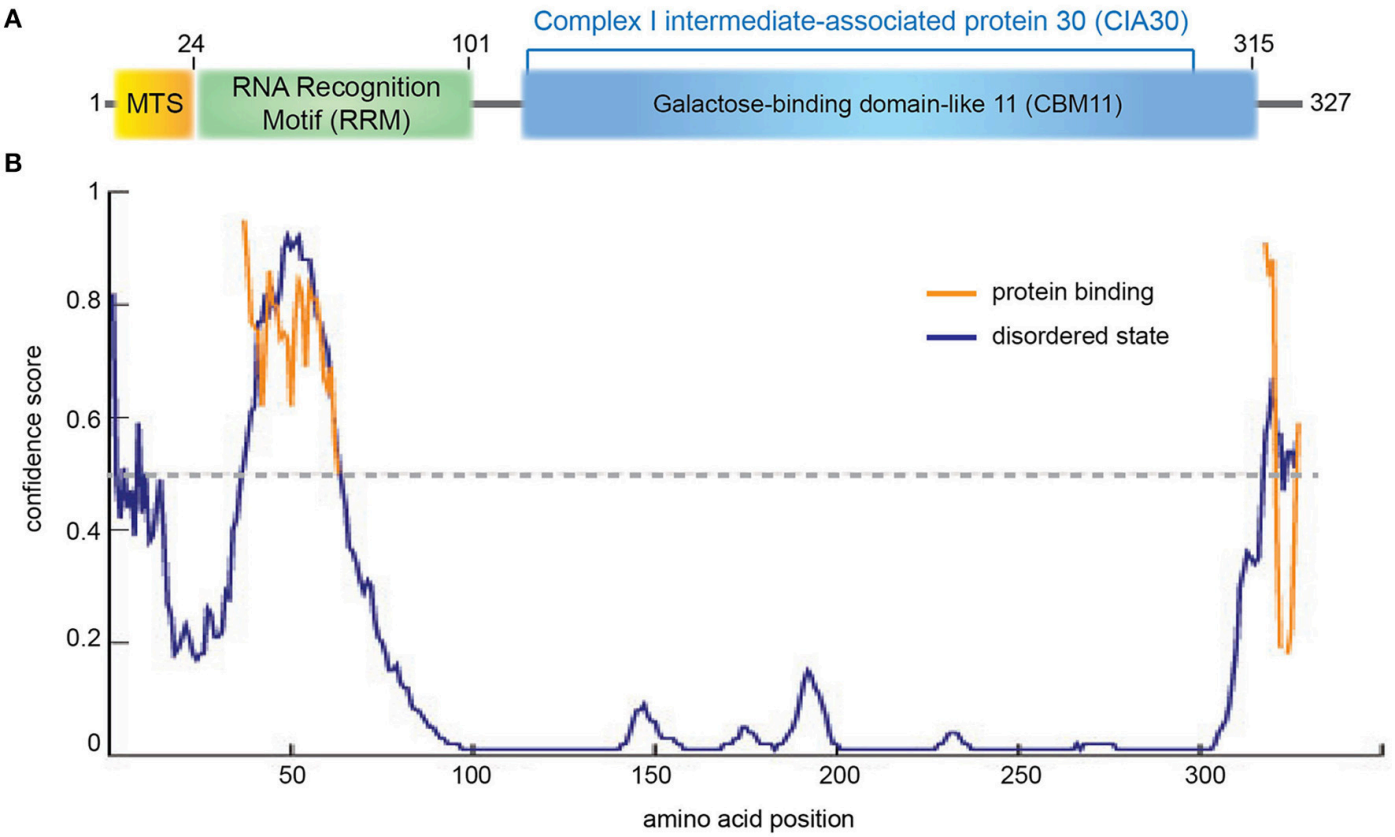
**NDUFAF1 protein domain organization. (A)** Human NDUFAF1 predicted domain organization. MTS: mitochondrial signal peptide (residues 1−24). RRM: predicted RNA recognition motif (residues 14−101) based on a BLAST conservation domain alignment [(Marchler-Bauer et al., 2015); *superfamily member cl17169, Pssm-ID:277499, E-value* = *5.72e-04*]. The C-terminal half of the protein belongs to the CIA30 family domain [(residues 125−298)*; superfamily member* cl21728, *Pssm-ID:272148, E-value* = *6.40e-55*], which is predicted to include a carbohydrate-binding module (CMB11) based on InterPro server (*ID:IPR005087*; Mitchell et al., 2015). **(B)** Prediction of disorder tendency of the full-length NDUFAF1 with PSIPRED server (Buchan et al., 2013). High-confidence protein binding sites are shown in orange lines.

NDUFAF1 (NADH dehydrogenase [ubiquinone] 1 alpha subcomplex assembly factor 1) is a mitochondrial protein comprising 327 amino acids including a predicted 24 residue N-terminal MTS (Figure 2A). NDUFAF1 interacts with nuclear- and mitochondrial-encoded CI subunits (Supplementary Table 1; Dunning et al., 2007). siRNA-mediated knockdown of NDUFAF1 results in decreased CI activity and levels, while overexpression of NDUFAF1 leads to an increase in the CI expression (Vogel et al., 2005). Based on these results, NDUFAF1 has been proposed to be a chaperone transiently interacting with CI intermediates (Kuffner et al., 1998; Vogel et al., 2007b) but the mechanistic details are still elusive.

NDUFAF1 has been identified as the human homolog of *N. crassa* CI intermediate-associated protein 30 (CIA30) and is moderately conserved among species, sharing 28% identity and exhibiting the highest homology in the C-terminal region of the protein (Vogel et al., 2005; Figure 2, Supplementary Figure 1). Interestingly, using structural profile-based homology searches, the C-terminal region of NDUFAF1 shows a high similarity to Galactose-binding domain-like proteins, in particular to the non-catalytic carbohydrate binding module family 11 (CBM11; Viegas et al., 2008; Figure 2, Supplementary Figure 1). These modules fold as a β-sandwich structure with a high degree of similarity between different family members despite an often low level of sequence similarity. They usually exist within large enzymes, being involved in recognizing the appropriate glucans for the catalytic domain and in localizing those domains onto the surface of the polysaccharide substrates (Elurbe and Huynen, 2016).

The N-terminal region of NDUFAF1 appears to contain a putative RNA recognition motif (RRM; Figure 2, Supplementary Figure 1; Marchler-Bauer et al., 2015). The RRM superfamily, also known as RBD (RNA binding domain) or RNP (ribonucleoprotein domain), is a highly abundant domain in eukaryotes and is found in proteins involved in post-transcriptional gene expression processes including mRNA and rRNA processing, RNA export, and RNA stability. This domain is generally 90 amino acids in length with a tertiary structure consisting of a four-stranded β-sheet packed against two α-helices. RRM usually interacts with single-stranded RNA, but is also known to interact with single-stranded DNA and proteins. RRM binds to a variable number of nucleotides, ranging from two to eight (Dreyfuss et al., 1988). In NDUFAF1 this domain is predicted to be highly disordered and to be involved in protein-protein interactions (Figure 2B). NDUFAF1 also contains putative phosphorylation sites such as Ser18 located in the leader sequence and Ser199 located in the central region (Supplementary Figure 1, *residues highlighted in pink*; Janssen et al., 2002). Interestingly, a cAMP-dependent protein kinase phosphorylation site, located on the 18 kDa subunit of CI, has been shown to be involved in the activation of the complex (Janssen et al., 2002).

#### ECSIT

**Figure 3:**
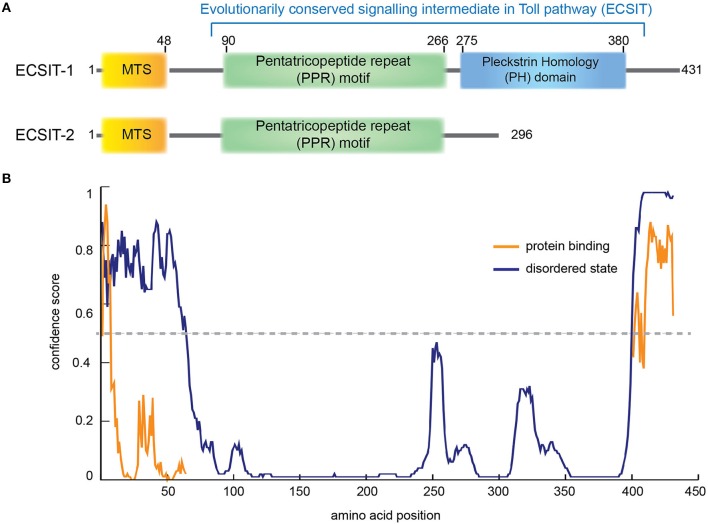
**ECSIT protein domain organization. (A)** Human ECSIT canonical isoform 1 and isoform 2 predicted domain organization, respectively. MTS: mitochondrial signal peptide (residues 1−48). PPR: pentatricopeptide repeat motif (central residues 90–266) based on a BLAST conservation domain alignment [*Pssm-ID:276811, Threshold Bit Score* = *35.6508* (Marchler-Bauer et al., 2015)]. The C-terminal part seems to fold like a pleckstrin homology (PH) domain (residues 275–380) based on the structure-based homology model server Phyre2 (Kelley et al., 2015). **(B)** Prediction of disorder tendency of the full-length ECSIT with PSIPRED server (Buchan et al., 2013). High-confidence protein binding sites are shown in orange lines.

ECSIT (Evolutionarily conserved signaling intermediate in Toll pathway) is an adapter protein of 431 amino acids (Kopp et al., 1999). There are 4 predicted isoforms but only isoforms 1 and 2 have been detected at protein level (Kopp et al., 1999; Xiao et al., 2003). Although ECSIT was initially identified as a cytoplasmic and nuclear signaling protein (Kopp et al., 1999; Xiao et al., 2003) an N-terminal MTS (first 48 amino acids) can direct ECSIT to mitochondria (Vogel et al., 2007a; Figure 3A).

The N-terminal region of ECSIT (spanning from residues 90 to 266 approximately) seems to be highly ordered (Figure 3B) and to contain pentatricopeptide repeats (PPR; Supplementary Figure 2). PPRs are 35-residue repeat motifs that form two anti-parallel α-helices organized into tandem repeats, typically binding single-stranded RNA in a sequence-specific and modular manner. They were first identified in plant organelles but an important role in mammalian mitochondrial gene regulation is now emerging (Rackham and Filipovska, 2012). PPRs are involved in many aspects of RNA metabolism such as maturation, translation and stabilizing organelle transcripts. Interestingly, PPR domain proteins (PTCDs) are predicted to be involved in the assembly of ETC complexes (Lightowlers and Chrzanowska-Lightowlers, 2008). Taken together, although no RNA binding for ECSIT nor NDUFAF1 has been reported, the fact that they both may contain an RNA binding domain suggests that the MCIA complex could also be involved in complex biogenesis by regulating the RNA processing of mitochondrial-encoded CI subunits (Rackham and Filipovska, 2012; Lightowlers and Chrzanowska-Lightowlers, 2013; Olahova et al., 2015).

The C-terminal domain of ECSIT shows a higher intrinsic disorder degree (Figure 3B) and only occurs in metazoa, indicating that ECSIT itself is also limited to this taxon, i.e., the filozoa (Elurbe and Huynen, 2016). Fold recognition suggests that this domain may be distantly homologous to the pleckstrin homology (PH) domain (Kelley et al., 2015). Despite minimal sequence homology, the three-dimensional structure is remarkably conserved among PH domains, with minimal secondary structure elements consisting of seven β-strands and one C-terminal α-helix (Rebecchi and Scarlata, 1998). The number and variety of host proteins with PH motifs is large but most of them can be grouped by function into a few classes: Ser/Thr protein kinases, Tyr protein kinases, small G-protein regulators, endocytic GTPases, adaptors, phosphoinositide metabolizing enzymes, and cytoskeletal associated proteins (Rebecchi and Scarlata, 1998). Many of them contain a catalytic domain (e.g., kinase) and other adaptor domains (e.g., SH2 or SH3) and they are often the targets for protein kinases (Rebecchi and Scarlata, 1998). Notably, ECSIT isoform 2 lacks this domain and seems to be involved in the BMP signaling pathway as a SMAD cofactor, required for normal embryonic development (Xiao et al., 2003).

ECSIT interacts with a ubiquitin ligase called TRAF6 (tumor necrosis factor receptor–associated factor 6) and it is involved in phosphorylation and activation of the nuclear factor NF-ƙB pathway in innate immunity (Kopp et al., 1999). Ubiquitination of ECSIT at residue K372 is critical for NF-ƙB binding activity and its translocation to the nucleus (Wi et al., 2014). Experimental evidence shows that the N-terminal region of ECSIT comprising residues 200–260 co-purifies with TRAF6 (Wi et al., 2014) suggesting that the ECSIT PPR domain might be involved in TRAF6-specific binding (Figure 3B). The C-terminal region of ECSIT (residues 260−431) specifically binds to the TGF-beta-activated kinase 1 (TAK1; Wi et al., 2014), which could indicate that the PH domain plays a role in the ECSIT-TAK1 binding to induce the activation of NF-ƙB-dependent gene expression (Supplementary Figure 2).

Mitochondrial ECSIT participates in CI assembly (Supplementary Table 2; Vogel et al., 2007a; Nouws et al., 2010; West et al., 2011). The knockdown of ECSIT in human cells reduces the levels of NDUFAF1 and results in impaired CI assembly and activity (Vogel et al., 2007a). In fact, the mitochondrial isoform of ECSIT was found in 500–600-kDa and 830-kDa assembly intermediates of CI and associated with NDUFAF1 (Vogel et al., 2007a). Furthermore, knockdown of ECSIT in cells results in disturbed mitochondrial function, supporting a role for ECSIT in linking the assembly of oxidative phosphorylation complexes with the inflammatory response (Vogel et al., 2007a).

#### ACAD9

**Figure 4:**
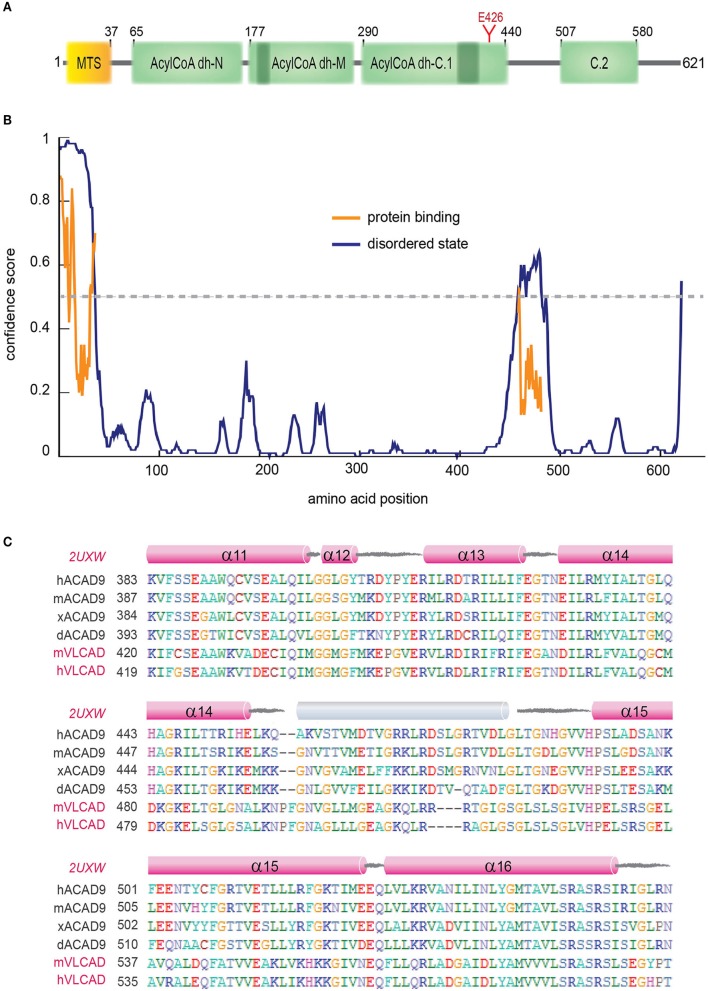
**ACAD9 protein domain organization. (A)** Human ACAD9 domain organization. MTS: mitochondrial signal peptide (residues 1–37). It contains three Acyl-CoA dehydrogenase/oxidase domains based on InterPro server (Mitchell et al., 2015): N-terminal (residues 65–175, *ID:IPR013786*); central (residues 177−277, *ID:IPR006091*); and a C-terminal split into two subdomains (C.1. residues 290–440 and C.2. 507–580, *ID:IPR009075*). Acyl-dehydrogenase catalytic residue E426 is shown in red. **(B)** Prediction of disorder tendency of the full-length ACAD9 with PSIPRED server (Buchan et al., 2013). High-confidence protein binding sites are shown in orange lines. **(C)** Multiple alignment of ACAD9 and VLCAD (highlighted in pink) orthologues around ACAD9 residues 383 to 561, based on CLUSTALX (Larkin et al., 2007) and edited with BioEdit (Hall, 2011). This regions shows the highest sequence variability between ACAD9 and VLCAD homologs and is predicted to correlate with the folding of an external 35 residue helix (shown in gray) that seems to be absent in the VLCAD crystal structure (PDB code 2UXW, McAndrew et al., 2008) and which might represent a specific interaction domain site unique for ACAD9. Residue numbering according to human ACAD9 sequence. h, human; m, mouse; x, African frog; d, zebrafish.

Acyl-CoA dehydrogenase 9 (ACAD9) is a 621 amino acid protein with an N-terminal MTS, three Acyl-CoA dehydrogenase domains, two conserved ACAD motifs and a potential N-glycosylation site (Figure 4A, Supplementary Figure 3). ACAD9 undergoes a mitochondrial processing, resulting in the cleavage of the first 37 amino acids from the precursor protein and leaving residue A38 as the N-terminal amino acid of the mature form of the enzyme (Figure 4). The ACAD family comprises mitochondrial flavoenzymes that catalyze the initial rate-limiting step of the fatty acid β-oxidation, which is one of the main energy-producing metabolic pathways in eukaryotes. While different dehydrogenases target fatty acids of varying chain length, all ACADs are mechanistically similar and use FAD as a required co-factor in addition to the presence of an active site glutamate in order for the enzyme to function. ACAD9 is very similar to its ancestor, the very long-chain acyl-CoA dehydrogenase (VLCAD), sharing 47% sequence identity and 67% similarity (Nouws et al., 2010). ACAD9 was originally annotated in vertebrates as a result from VLCAD gene duplication. However, it is has also been found in non-vertebrate metazoa (Elurbe and Huynen, 2016). The amino acid sequence of ACAD9 contains the two conserved dehydrogenase signatures (Supplementary Figure 3) and an important active site residue (E426; Figure 4A). These features confer some residual acyl dehydrogenase activity, suggesting that the enzymatic activity is a rudiment of the VLCAD gene duplication event (Nouws et al., 2014). However, unlike VLCAD, ACAD9 seems critical for oxidative phosphorylation (Nouws et al., 2014) and plays a key role as a CI assembly factor, as shown by different approaches (Nouws et al., 2010). Indeed, it binds ECSIT and NDUFAF1 (Supplementary Table 3) and mutations on ACAD9 cause CI deficiencies (Table 2; Nouws et al., 2014). Knockdown of ACAD9, NDUFAF1, or ECSIT in cultured cells determines the decrease of all three proteins and of CI holo-complex as well (Gerards et al., 2011).

**Table 2:** **Currently identified pathological mutations in CI assembly factors causing CID**.

**Assembly factors**	**UniProt entries**	**Annotated mutations**	**Disease phenotypes**	**References**
NDUFAF1	Q9Y375	H92R, T207P, K253R, R211C, G245R	LS, L, HC	Dunning et al., 2007; Fassone et al., 2011; Dewulf et al., 2016
NDUFAF2	Q8N183	M1L, W3^STOP^, Y38^STOP^, R45^STOP^, W74^STOP^, I35SfsX17^a^, A73GfsX5^a^	LS, L	Ogilvie et al., 2005; Barghuti et al., 2008; Hoefs et al., 2009; Janssen et al., 2009; Calvo et al., 2010; Herzer et al., 2010; Ghaloul-Gonzalez et al., 2016
NDUFAF3	Q9BU61	M1T, G77R, R122P	FLA	Saada et al., 2009
NDUFAF4	Q9P032	L65P	LS, L	Saada et al., 2009
NDUFAF5	Q5TEU4	L159F, L229P	LS	Sugiana et al., 2008; Gerards et al., 2010
NDUFAF6	Q330K2	Q99R	LS, L	Pagliarini et al., 2008
NDUFAF7	Q7L592	n.d.	n.d.	
FOXRED1	Q96CU9	Q232^STOP^, R352W, V421M, N430S	LS, L	Calvo et al., 2010; Fassone et al., 2011; Zurita Rendón et al., 2014
NUBPL	Q8TB37	G56R, D105Y, L193F, E223AfsX4^a^, 240-kb deletion (exons 1–4); 137-kb duplication (exon 7)	LS, L	Calvo et al., 2012; Tucker et al., 2012; Kevelam et al., 2013
TMEM126B	Q8IUX1	G212V, N134IfsX2^a^	CID	Alston et al., 2016
ACAD9	Q9H845	F44I, E63^STOP^, F120SfsX9^a^, R127Q, A170V, G172R, S268F, T243R, R266Q/W, L314P, A326P, F339V, E413K, R414S, R417C, R433Q, R469W, R518C, R532W/Q, V546L, L558PfsX45^a^, H563A	LS, L, HC	Scholte et al., 1995; Haack et al., 2010; Nouws et al., 2010; Gerards et al., 2011; Collet et al., 2016; Dewulf et al., 2016; Leslie et al., 2016; Pronicka et al., 2016
ECSIT	Q9BQ95	n.d.	n.d.	
TIMMDC1	Q9NPL8	n.d.	n.d.	

*LS, Leigh syndrome; L, leukodystrophy; HC, hypertrophic cardiomyopathy; FLA, fatal lactic acidosis, encephalopathy; CID, complex I deficiencies; n.d., not determined or not (yet) associated with disease*.

a *fsX = frameshift*.

A homology model of the structure of an ACAD9 dimer based on the VLCAD crystal structure is shown in Figure 4B (Nouws et al., 2010). Unlike other ACADs, human VLCAD, and ACAD9 contain an extension of the C-terminus that has been suggested to be involved in intra-mitochondrial membrane binding (Swigonova et al., 2009). The homodimer model further indicates that sequence differences in ACAD9 are associated with two solvent exposed 35 amino acid-long α-helices, which might represent a new interaction domain important for its acquired role in respiratory complex assembly in vertebrates (Supplementary Table 3, Figure 4C; Nouws et al., 2010; Scheffler, 2010; Mick et al., 2012). Interestingly, this region is predicted to be a high-confidence protein binding site (Buchan et al., 2013; Figure 4B). Furthermore, although the role of the FAD cofactor in CI assembly is unclear, it may function as a chemical chaperone and improve specific ACAD9 folding (Nouws et al., 2014).

#### TMEM126B

Transmembrane protein 126B (TMEM126B) is an integral component of the inner mitochondrial membrane and comprises 230 residues folded in 4 transmembrane α-helices (Heide et al., 2012). TMEM126B is found exclusively in mammals and likely resulted from a segmental duplication of TMEM126A, an inner mitochondrial membrane protein of unknown function (Elurbe and Huynen, 2016). Although the molecular function of TMEM126B has not been clarified yet, it has been recently discovered that it is required for the CI assembly (Supplementary Table 4; Heide et al., 2012). One of the critical functions of this membrane protein might be the recruitment of the hydrophilic MCIA components to constitute a functional MCIA complex (Vartak et al., 2014). Indeed, NDUFAF1, ECSIT, and ACAD9 are not recruited to the membrane in case of lack of TMEM126B, leading to an MCIA complex misassembly (Vartak et al., 2014). Furthermore, depletion of TMEM126B leads to accumulation of the Q module-ND1 intermediate (Guarani et al., 2014) and severely impairs mitochondrial respiration (Heide et al., 2012).

#### TIMMDC1

The translocase of inner mitochondrial membrane domain-containing 1 (TIMMDC1) protein contains 285 residues embedded in four transmembrane α-helices. It belongs to the TIM17-TIM22-TIM23 (translocases of the inner mitochondrial membrane) domain family, which are membrane-embedded multi-protein complexes that mediate the transport of nuclear-encoded proteins across and into inner mitochondrial membranes (Kurz et al., 1999; Chacinska et al., 2005). Recent data show that the transmembrane protein TIMMDC1 is also physically associated with the MCIA complex and functions in CI assembly (Supplementary Table 5; Guarani et al., 2014). Depletion of TIMMDC1 in tissue culture cells leads to accumulation of CI subcomplexes similar to MCIA factor depletion effects, resulting in impaired CI activity and cellular respiration and a decreased stability of several CI subunits (Guarani et al., 2014).

### The molecular assembly of the MCIA complex

Overall, the available data show that depletion of the MCIA factors NDUFAF1, ECSIT, or ACAD9 leads to the accumulation of the 370-kDa assembly intermediate from the CI membrane arm, which contains mtDNA-encoded subunits with about 20 transmembrane α-helices (Andrews et al., 2013). How the MCIA factors function is not known yet, but they may stabilize this subcomplex and promote its incorporation to the other subcomplexes to build up the complete holo-enzyme. However, the MCIA complex could also have a more general function, regulating CI assembly at the translation level by the induction and stabilization of the transcripts of mtDNA-encoded subunits when required (Supplementary Tables 2–5). The fact that the sequential assembly pathway of MCIA factors is still incomplete suggests that additional extrinsic and/or intrinsic assembly factors remain to be identified (Table 1; Andrews et al., 2013).

**Figure 5:**
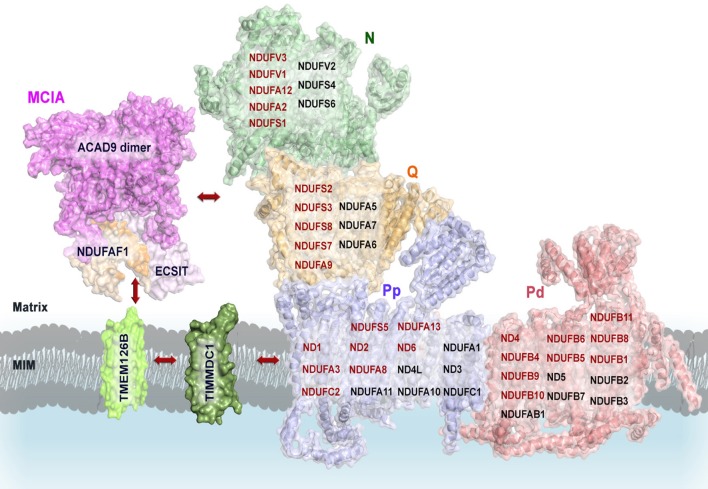
**Proposed model of the molecular assembly of the MCIA complex and its role in the assembly of the CI**. Complete view of the assembly pathway indicating the steps where MCIA assembly factors are involved (see text for details). Representation of mammalian mitochondrial CI model is based on the bovine heart cryo-EM structure [(Vinothkumar et al., 2014); *PDB 4UQ8*]; ACAD9 on the homology model proposed by (Nouws et al., 2010); NDUFAF1, ECSIT, TIMMDC1, and TMEM126B on putative homology folding. The matrix-hydrophilic functional modules are colored in green (N) and orange (Q), respectively. Membrane-embedded modules, in blue (Pp, Pump proximal), and in salmon (Pd, Pump distal). CI subunits with reported interactions with MCIA factors are shown in red. MIM, mitochondrial inner membrane.

The homology model of ACAD9 dimer provides initial insights into the molecular assembly of the MCIA complex (Figure 5). In a first step, the newly proposed interaction domain of ACAD9 homodimer could bind to ECSIT and NDUFAF1, respectively, forming a tetrameric complex that would then interact with the membrane assembly factor TMEM126B to anchor the MCIA complex onto the membrane (Nouws et al., 2010). Indeed, it has been suggested that TMEM126B would insert hydrophobic proteins into the 370-kDa subcomplex to subsequently associate with the 315-kDa subassembly and to finally constitute a subcomplex of 550 kDa (Andrews et al., 2013). Furthermore, TIMMDC1 could be involved in the transfer of newly imported nuclear-encoded subunits and MCIA factors as well, similarly to the other family member TIM21 in CIV assembly (Mick et al., 2012). TIMMDC1 would then engage with assembly factors after import and, as it has been recently speculated, it would also function as a membrane anchor to assemble additional components of the MCIA complex, together with both the Q module components and the major membrane arm, and to organize intermediates into a productive assembly pathway (Guarani et al., 2014). During the final stages of CI assembly, these factors would dissociate, since these proteins are not found associated with the mature holo-enzyme (Mckenzie and Ryan, 2010).

Nonetheless, considering that protein disorder prediction algorithms are not 100% accurate, especially for transmembrane topology prediction (Buchan et al., 2013), experimental data are essential for a complete understanding of the MCIA complex assembly and the underlying molecular mechanisms of the CI assembly process at the structural level. The development of reconstituted systems at high resolution by X-ray crystallography or electron microscopy will thus enable to unravel the protein complex organization down to the atomic detail.

## Defects in complex I assembly result in neurodegenerative diseases

As described earlier, CI acts as metabolism hub essential in cellular energy production, but it is also the major source of ROS production. Redox signals can mediate through cysteine oxidation, namely S-oxidation, S-glutathionylation, and S-nitrosylation, which have been shown to also regulate CI activity (Bak and Weerapana, 2015; Mailloux, 2015). Hence, even subtle defects in CI assembly or function are directly linked to oxidative stress and ultimately to mitochondrial dysfunction and disease. When ROS production overwhelms the endogenous antioxidant systems this can lead to oxidative damage in mtDNA, membranes or proteins, impairing cellular functions such as ATP and heme synthesis, fatty acid oxidation, or the urea and tricarboxylic acid cycles. Mitochondrial ROS damage can also lead to cytochrome-*c* release to the cytosol by mitochondrial outer membrane permeabilization and thereby trigger apoptosis through the activation of the caspase cascade (Jeong and Seol, 2008). Neuronal cells are particularly susceptible to ROS-induced damage because they mainly rely on oxidative metabolism for ATP generation, in contrast to glial cells, which are highly glycolytic (Belanger et al., 2011). However, there are metabolic interactions from astrocytes to neurons that appear to play an important role in the control of neuronal activity and excitability (Belanger et al., 2011).

Mitochondrial integrity also declines as a consequence of aging. Interestingly, some of the functional impairments seem to be correlated with increasing oxidative stress derived from defects in the OXPHOS system. Furthermore, age-related neurodegenerative diseases (NDs) may exacerbate the oxidative damage. Indeed, mitochondrial dysfunction represents a common pathogenic mechanism in NDs like Alzheimer's disease (AD), Parkinson's disease (PD), amyotrophic lateral sclerosis, Huntington's disease, and prion diseases (Beal, 2002; Lin and Beal, 2006; Tillement et al., 2011; Federico et al., 2012; Schapira, 2012; Butterfield et al., 2016). In particular, CI activity is reduced with aging, as demonstrated in primate brains and in long-lived mice models (Bowling et al., 1993; Miwa et al., 2014). Typically, genetic defects in CI and its assembly factors account for a heterogeneous group of fatal disorders collectively known as complex I deficiencies (CID). However, recent clinical and experimental studies also indicate a possible link between CI dysfunction and the pathogenesis of AD or PD, which have accelerated the interest toward investigations into CI biology as a promising therapeutic target for NDs (Eckert et al., 2010; Winklhofer and Haass, 2010).

### Neurodegeneration associated with complex I deficiencies

CID is the most prevalent genetic defect in mitochondrial energy metabolism, accounting for approximately a third of the OXPHOS disorder cases (Kirby et al., 1999). CID is characterized by marked genetic, tissue, and organ specific heterogeneity with often overlapping clinical phenotypes. The majority of the affected patients also presents Leigh syndrome (LS): a fatal, incurable, rapidly progressive neurodegenerative disorder typically occurring within the first weeks/months after birth and with an incidence of at least 1 case out of 40,000 live births (Darin et al., 2001). Neuropathologically, LS features bilateral symmetrical necrotic lesions characterized by spongiosis, neuronal loss, astrocytosis, and capillary proliferation; clinical signs and symptoms include muscular hypotonia, developmental delay, abnormal eye movements, respiratory abnormality, seizures, and ataxia (Leigh, 1951; Rahman et al., 1996). Additional CID clinical phenotypes have been associated with other childhood ND, including Leigh-like syndrome, leukoencephalopathy, MELAS (mitochondrial encephalomyopathy, lactic acidosis and stroke-like episodes), and NARP (neuropathy, ataxia and retinitis pigmentosa) syndromes (Fassone and Rahman, 2012; Koopman et al., 2016).

Mutations in six mtDNA-encoded (ND1 to 6) and thirteen nuclear-encoded (NDUFS1 to 8; NDUFV1; NDUFA1, 2, 9, 10, and 12) CI subunits have been correlated to LS (Rodenburg, 2016). Early investigations on CID patient-derived fibroblasts showed partial or incomplete CI assembly and diminished catalytic activity (Verkaart et al., 2007a), which was inversely correlated with ROS production in mitochondria-enriched fractions (Koopman et al., 2007; Verkaart et al., 2007b). In addition, CID patient fibroblasts showed a significantly depolarized mitochondrial membrane, which in turn caused a dramatic effect on ATP production and protein transport across mitochondrial membranes (Komen et al., 2007; Distelmaier et al., 2009a,b). Finally, aberrations in CI activity have an impact in the calcium homeostasis, a crucial stimulus for activating ATP production via OXPHOS, as observed in CID derived cells (Willems et al., 2008). Nevertheless, while much progress has been made in elucidating the structure of mammalian CI, the molecular mechanisms underlying CID are still poorly understood.

Moreover, clinical studies have reported that only about 33% of the CID cases are associated with specific genetic defects in the 44 CI subunits (Calvo et al., 2010), revealing that assembly factors may account for the remaining CID cases (Nouws et al., 2012). Interestingly, the first evidence of the role of assembly factors in CID comes from the identification of pathological mutations in genes encoding for MCIA components and other CI assembly factors, most of them linked to CID but with an additional broad spectrum of disease phenotypes, such as LS, leukodystrophy, MELAS and NARP syndromes (Table 2; Nouws et al., 2012; Rodenburg, 2016; Wu et al., 2016). Along this line, several ACAD9 mutations are associated to CID (Haack et al., 2010; Rodenburg, 2016) and indeed, stabilization of ACAD9 might represent a therapeutic approach for treating CID, as shown by a riboflavin treatment (the central component of FAD cofactors) where CI activity increased and clinical conditions of ACAD9-defective patients improved (Gerards et al., 2011). Some other MCIA factors may display a pleiotropic role in cells, such as ECSIT, which was identified as an inter-pathway protein node interacting with Aβ-producing enzymes (Soler-Lopez et al., 2011), suggesting a link between CI assembly/stability and AD pathogenesis. Upon inflammation or Aβ insult, ECSIT may expand the signaling of the immune response to the inner-mitochondrial level, e.g., by stabilizing CI to ensure mitochondrial physiology, or induce apoptosis when repair failure (Soler-Lopez et al., 2012). Recently, two biallelic mutations have been identified in TMEM126B, which cause CID with a broad disease phenotype. Interestingly, patient-derived cell lines show markedly CI assembly defects (like accumulation of intermediates) and decreased levels of the remaining MCIA components: ECSIT, ACAD9, and NDUFAF1 (Alston et al., 2016).

Notably, recent investigations have revealed that around 40% of CID cases are not associated with mutations in CI subunit or assembly factor encoding genes, suggesting that other yet unknown factors may affect the proper function of the ETC (Taylor et al., 2014). These findings have prompted the interest toward the identification of novel assembly factors in order to provide a genetic explanation and potential therapeutic strategies for those CID cases not directly linked to CI subunit mutations. To achieve this goal, genome-wide sequencing, proteomic and structural approaches will be highly relevant in the identification and characterization of CID-associated genes whose physiological roles are still undetermined.

### Complex I dysfunction and parkinson's disease

CI dysfunction also seems to be critical in PD pathogenesis, correlated with the degeneration of dopaminergic neurons in the *substantia nigra pars compacta* (Schapira et al., 1990; David et al., 2005; Keeney et al., 2006; Gatt et al., 2016). One of the PD pathological hallmarks is the presence of intracellular inclusions called Lewy bodies that consist of aggregates of the presynaptic protein α-synuclein (Dawson and Dawson, 2003). Besides α-synuclein, several proteins have also been associated with PD, some of which (e.g., DJ-1, PINK1, Parkin, HTRA2) localize in the mitochondria and gain toxic functions due to mutations that may lead to mitochondrial dysfunction (Federico et al., 2012). In fact, CI defects and increased oxidative damage are consistent features of both sporadic (idiopathic) and familial (genetic) PD forms. Decrease of CI activity has been reported in the *substantia nigra* and in the cortex of PD patients (Haelterman et al., 2014).

Early experiments studying CI induced inhibition by agrochemicals, such as rotenone or 1-methyl-4-phenyl-1,2,3,6-tetrahydropyridine (MPTP), showed a correlation with PD symptoms like the formation of α-synuclein containing inclusions, bioenergetics defects and ROS overproduction (Liang et al., 2007; Sherer et al., 2007). Although it is plausible that a chronic exposure to CI inhibitors might contribute to the development of sporadic PD, it is unlikely that toxin exposure accounts for CI defects in the general population. An alternative hypothesis proposes that inherited or somatic mutations in mtDNA might account for CI defects triggering oxidative stress and PD, experimentally supported by PD cytoplasmic hybrid (i.e., cybrid) cell lines showing higher ROS levels (Swerdlow et al., 1996; Gu et al., 1998). Different studies have reported somatic mutations in ND4 and ND5 CI subunits in PD patients (Simon et al., 2004; Parker and Parks, 2005). Furthermore, α-synuclein contains an MTS that enables an eventual translocation to the inner mitochondrial membrane (Martin et al., 2006). How mitochondrial localization of α-synuclein affects mitochondrial function in human brain has not been addressed in detail, although independent observations propose a role for α-synuclein in maintaining the OXPHOS physiological functions (Ellis et al., 2005; Devi et al., 2008). In transgenic (tg) mice, over-expression of α-synuclein impairs mitochondrial function, increases oxidative stress and enhances the *substantia nigra* pathology induced by MPTP (Song et al., 2004). Interestingly, *in vivo* data using human fetal dopaminergic primary neuronal cells and PD brain-derived tissues (*striatum* and *substantia nigra*) have shown a progressive accumulation of α-synuclein in the mitochondria resulting in impaired CI functioning and increased oxidative stress. Remarkably, α-synuclein has been found in direct association with the ~600-kDa subcomplex and the holo-CI in mitochondria derived from PD patients (Devi et al., 2008). These results suggest that α-synuclein accumulation in the mitochondrial matrix may also affect the proper assembly of CI and lead to mitochondrial dysfunction. Other studies have shown that some core (ND4, ND5, NDUFS1, NDUFS2, and NDUFV1) and accessory (NDUFB5, NDUFB6, and NDUFB7) subunits are oxidatively damaged in PD brains, resulting in CI misassembling and functional impairment (Keeney et al., 2006).

Overall, these findings support the assumption that CI misassembly may play a role in PD onset and progression. However, the molecular mechanisms contributing to the instability of CI assembly, loss of bioenergetic functions and oxidative stress are yet unclear. It is tempting to speculate that abnormal α-synuclein accumulation in mitochondria, mutations, or polymorphisms in PD-associated mitochondrial genes may lead to defects in the CI assembly process, which eventually plays a central role in PD pathology.

### Complex I dysfunction and Alzheimer's disease

AD is the most prevalent form of ND, characterized by a progressive memory loss and impairment of cognitive abilities (Duyckaerts et al., 2009; Hyman et al., 2012). At the neuropathologic level, it reveals the presence of amyloid plaques and neurofibrillary tangles (NFT) as the final result of misfolding and aggregation of amyloid-β (Aβ) and tau proteins. Aβ is the cleavage product of a much larger protein, the Amyloid Precursor Protein (APP), by α-, β-, and γ-secretases (Selkoe, 2001). Although different Aβ truncated species can be generated during the cleavage process (Willem et al., 2015), Aβ_1−42_ (i.e., the 42-amino acid form of Aβ) is the most neurotoxic peptide and represents the majority of Aβ deposits in AD brain (Welander et al., 2009). Aβ deposition leads to the formation of plaques in neuronal (neurites and synaptic terminals) and glial (astrocytes and microglia) cells. NFTs are intracellular aggregates of abnormal hyper-phosphorylated tau that form cytoplasmic fibrils (Braak et al., 1994). While the neurotoxicity of Aβ and tau misfolding, aggregation and spreading have been deeply investigated, much less is known about the early molecular events underlying AD pathogenesis. Intraneuronal Aβ accumulation has emerged as one of the main causative effects of synaptic damage and cognitive decline in AD (LaFerla et al., 2007). According to the AD mitochondrial hypothesis, defects in mitochondrial metabolism and particularly in the ETC may play a role during the early stage of AD pathogenesis (Valla et al., 2001). Aβ has been detected in mitochondria from both tg mice and murine cell lines expressing human mutant APP, displaying increased ROS production, decreased cytochrome oxidase activity, morphological mitochondrial alteration, and apoptosis (Manczak et al., 2006; Cha et al., 2012). Furthermore, Aβ has also been associated with mitochondria from AD patients, showing mitochondrial fragmentation in various brain regions (Reddy et al., 2010). As extensively reviewed, Aβ accumulation in the mitochondria leads to different mitotoxic events, such as permeabilization of membranes, reduction of respiratory function, and disturbance of the mitochondrial calcium homeostasis (Swerdlow et al., 2010; Moreira et al., 2010a; Tillement et al., 2011). Among these mechanisms, impaired OXPHOS functions have been frequently observed in AD patients and in different AD tg mouse and cellular models (Hroudova et al., 2014).

Clinical investigations have reported impairment of CI activity and reduced level of its subunits in multiple zones of *post mortem* AD brains (Mutisya et al., 1994; Aksenov et al., 1999; Kim et al., 2000, 2001), as well as in other tissues such as platelets (Cardoso et al., 2004). A number of proteomic studies on AD tg mouse models have provided clear evidence of the involvement of Aβ and tau proteins in CI defects and consequent mitochondrial dysfunction (David et al., 2005; Rhein et al., 2009; Chou et al., 2011; Zhang et al., 2015). Interestingly, tg mice overexpressing the tau P301L mutation (i.e., causing hyperphosphorylated tau accumulation and NFT) exhibit reduced CI activity and, with age, impaired mitochondrial respiration and ATP synthesis. Furthermore, isolated cortical brain cells from this mouse model display modified lipid peroxidation, increased ROS production and altered mitochondrial membrane potential after Aβ insults (David et al., 2005). This finding has led to propose a tau and Aβ synergistic contribution to mitochondrial pathology by inhibiting ETC -particularly CI- and inducing ROS-derived apoptosis. Consistent with this hypothesis, deregulation of CI and CIV has also been observed in triple-tg AD mice, (co-expressing AD-linked mutations in presenilin 2, APP, and tau proteins; Rhein et al., 2009). Importantly, in 8-month old tg mice (i.e., prior the appearance of the disease symptoms) deregulation of CI seems to be tau-dependent, whereas that of CIV is Aβ-dependent. However, with aging and especially in the presence of both Aβ plaques and NTF, defects on CI and CIV become more marked resulting in a significant decrease of respiratory parameters, reduced mitochondrial membrane potential and increased ROS level, providing further evidence to a synergistic detrimental effect of Aβ and tau on ETC (Rhein et al., 2009). Furthermore, in support of the central role of CI in AD pathogenesis, recent data suggest that CI-derived ROS contributes to amyloidogenic APP processing (Leuner et al., 2012; Tamagno et al., 2012; Bobba et al., 2013). Taken together, Aβ- or tau-mediated CI impairment result in a vicious cycle inducing electron leakage from the ETC leading boosting mitochondrial dysfunction and oxidative stress. Notably, pathological mechanisms underlying AD pathogenesis share common features with LS and the other CID forms previously described. A link between CID and AD has been recently established with the identification of a naturally occurring mutation, S339G, in the ND4 core subunit of CI of a premature aging mouse strain, SAMP8 (Imanishi et al., 2011). SAMP8 mice develop early learning and memory deficits together with other characteristics similar to those seen in AD, including ROS overproduction and Aβ plaques with aging (Morley et al., 2000, 2012). Interestingly, an adjacent human mutation, R340H, in the ND4 gene also results in CID with associated LS, Leber hereditary optic neuropathy and late onset MELAS syndrome, characterized by increased ROS production and altered CI assembly (Wong et al., 2002; Deschauer et al., 2003), prompting a new investigative field for the development of therapeutic approaches targeting mitochondria for the treatment of AD and CID (Friedland-Leuner et al., 2014).

## Perspectives in mitochondrial neurobiology

Emerging evidence suggest a pivotal role of mitochondrial dysfunction in the pathogenesis of major neurodegenerative disorders. In particular, CID is the most common OXPHOS disorder in humans and defects in CI assembly process are often detected. From a clinical point of view, mitochondrial biology holds the promise to provide novel insights into the pathogenesis of several NDs. Different proteomic investigations on mitochondrial proteins differentially expressed in CID (Pagliarini et al., 2008; Alston et al., 2016), AD (Sultana et al., 2011), and PD (Henchcliffe and Beal, 2008) suggest that proteins involved in CI assembly and functioning might be novel targets for biomarker identification, disease progression and ultimately, therapeutic approaches. Several drug discovery studies are under development to boost mitochondrial health or tune up the mitochondrial power engine to compensate for damaged or interrupted neuronal power failure upon neuronal stress (Moreira et al., 2010b). Novel therapeutic strategies will thus enable mitochondria to better cope with oxidative stress, excitotoxicity, and other neuronal stresses, as well as maintain efficient respiratory function in neurons. However, the drugs tested in most of the current trials do not target mitochondria but monitor mitochondrial function as an indicator of indirect effects of the treatments (Wang et al., 2016). There is increasing evidence that a fully assembled and stable CI is key in various pathways. Therefore, a better understanding of the regulatory mechanisms underlying CI assembly will unveil how mitochondria malfunction affects metabolic reprogramming and neuronal integrity. To this end, the molecular characterization of existing and novel assembly factors emerges as a promising approach for mitochondria-targeted therapeutics, the so-called mitochondrial medicine (Weissig et al., 2004), and for specific discrimination among ND disorders, with CI assembly factors gaining a new and exciting dimension.

## Conclusions

Despite the critical importance of CI in energy production and disease, many aspects of its assembly and activity are still poorly understood, since they require a stepwise coordination of different processes and components that are tightly interconnected. Clinical data have enabled the identification of CI subunits and assembly factors implicated in neurodegeneration, but they provide a limited understanding. Furthermore, other yet unknown factors may be involved in the proper function of the ETC. In this review, we have presented the current knowledge of CI structure and its assembly factors, with a particular focus on neurodegenerative disorders. Pathological mutations in CI assembly factors seem to affect the functional integrity of the holo-enzyme, although they have not been investigated at the molecular level yet. Therefore, structural studies on such proteins together with *in vivo* experiments will help us to elucidate the dynamics of CI biogenesis and will thus contribute to a better understanding of the altered mitochondrial pathways involved in neuronal death. These efforts will provide unique insights and will have a major contribution in the quest for novel disease-modifying approaches, where CI assembly factors might play a central role.

## Author contributions

GG and MS conceived the review. GG, RB, SA, SP, and MS wrote the manuscript. GG and MS edited the final version of the review.

### Conflict of interest statement

The authors declare that the research was conducted in the absence of any commercial or financial relationships that could be construed as a potential conflict of interest.

## Abbreviations

OXPHOS: oxidative phosphorylation system
CI: complex I
ETC: electron transfer chain
ROS: reactive oxygen species
mtDNA: mitochondrial DNA
FAD: flavine adenine nucleotide
MTS: mitochondrial targeting sequence
MCIA: mitochondrial CI assembly
ND: neurodegenerative diseases
AD: Alzheimer's disease
PD: Parkinson's disease
CID: complex I deficiencies
LS: Leigh syndrome
MELAS: mitochondrial encephalomyopathy, lactic acidosis and stroke-like episodes syndrome
NARP: neuropathy, ataxia, and retinitis pigmentosa syndrome
tg mice: transgenic mice
NFT: neurofibrillary tangles
Aβ: amyloid-β peptide
APP: amyloid precursor protein.
