# Supplementary material for: Dynamics of Human Mitochondrial Complex I Assembly: Implications for Neurodegenerative Diseases
Source: Front Mol Biosci. 2016 Aug 22;3:43. doi: 10.3389/fmolb.2016.00043 (PMC4992684; doi:10.3389/fmolb.2016.00043)
Supplement: Supplementary file 1 [file Presentation1.PDF]

Supplementary Material

Dynamics of Human Mitochondrial Complex I Assembly: Implications for Neurodegenerative Diseases

Gabriele Giachin, Romain Bouverot, Samira Acajjaoui, Serena Pantalone & Montserrat Soler-Lopez*

*** Correspondence:** Corresponding Author: montserrat.soler-lopez@esrf.fr

1 Supplementary Figures and Tables

1.1 Supplementary Figures

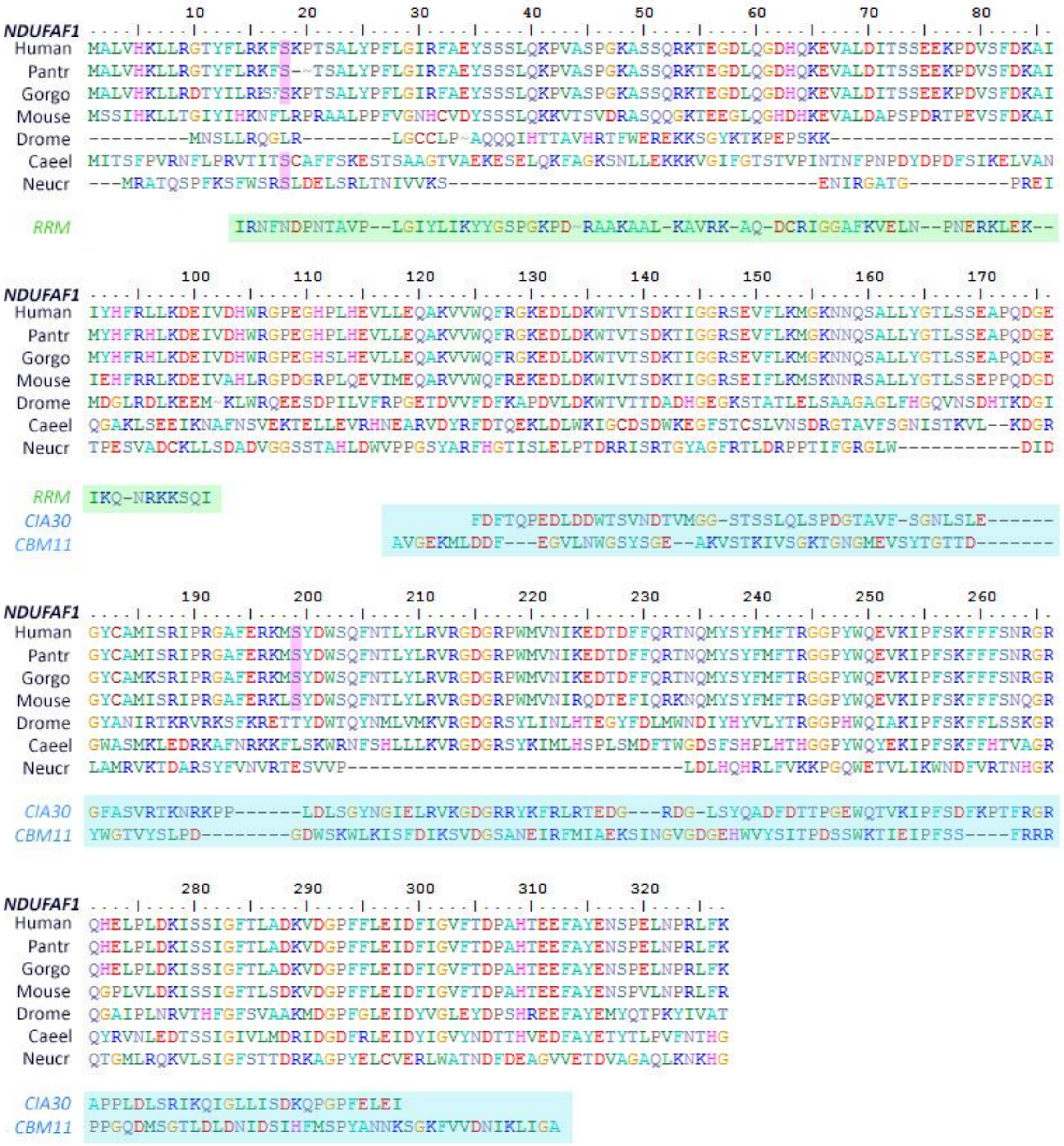

Supplementary Figure 1. NDUFAF1 domain conservation across species. Multiple alignment of NDUFAF1 orthologues based on CLUSTALX (Larkin et al. 2007) and edited with BioEdit (Hall 2011). Consensus sequences of potential conserved domains were aligned to the NDUAF1-sequence conservation alignment profile (RRM highlighted in green; CBM11 and CIA30 in blue). Putative phosphorylation sites are highlighted in purple. Residue numbering according to human NDUFAF1 sequence (in bold). *Pantr: chimpanzee; Gorgo: gorilla; Drome: fruit fly; Caeel: roundworm; Neucr: fungus*.

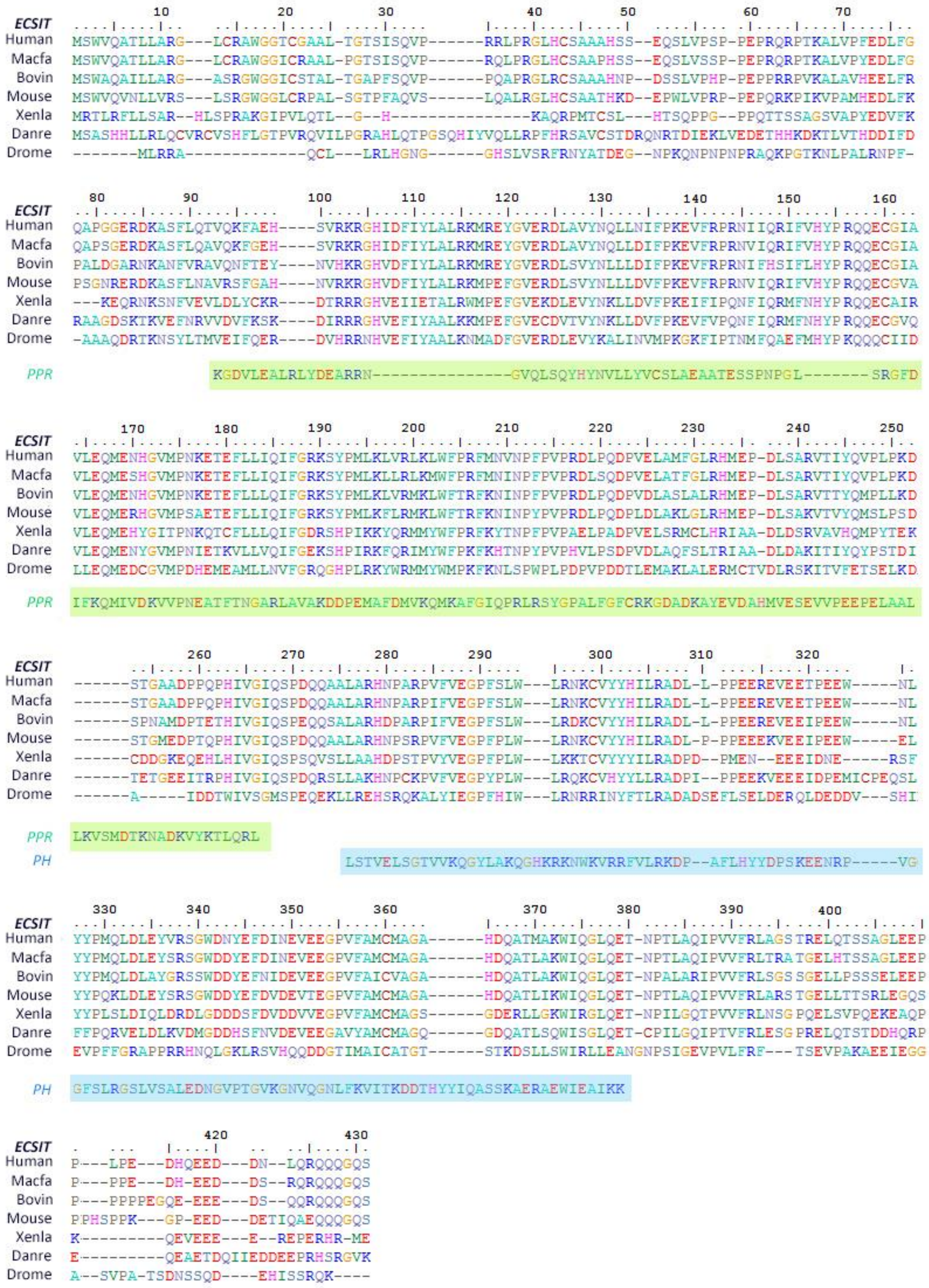

Supplementary Figure 2. ECSIT domain conservation across species. Multiple alignment of ECSIT orthologues based on CLUSTALX (Larkin et al. 2007) and edited with BioEdit (Hall 2011). BLAST conservation domain alignment predicted the presence of a RRM domain. Consensus sequences of PPR and PH domains were aligned to the conservation alignment profile, respectively (PPR highlighted in green; PH in blue). Residue numbering according to human ECSIT sequence (in bold). *Macfa: macaque; Bovin: bovine; Xenla: African frog; Danre: zebrafish; Drome: fruit fly.*

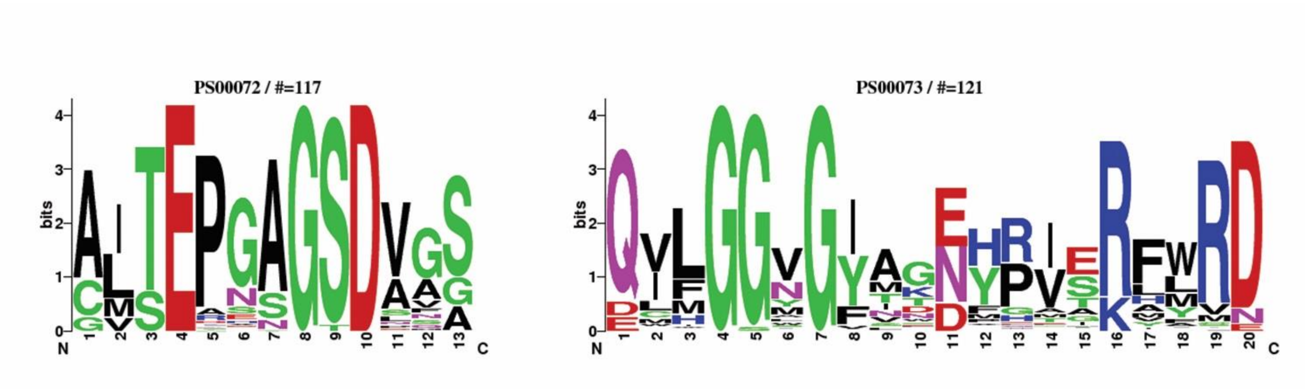

Supplementary Figure 3. ACAD9 conserved motifs. Consensus sequence in logo format of the two ACAD family conserved signatures using ScanPROSITE server (ACYL_COA_DH_1 PS00072, 117 hits, ACAD9 residues 179-191; ACYL_COA_DH_2 PS00073, 123hits, ACAD9 residues 399-418)(de Castro et al., 2006).

1.2 Supplementary Figures

Supplementary Table 1. NDUFAF1 protein interacting ETC partners. *Interactions curated by Intact (Orchard, 2014) and Biogrid (Chatr-aryamontri et al., 2015)*

Interactor	Description	ETC Function	References
ACAD9	Acyl-CoA dehydrogenase family member 9, mitochondrial	MCIA factor	Guarani et al. 2014
ECSIT	Evolutionarily conserved signaling intermediate in Toll pathway, mitochondrial	MCIA factor	Vogel et al. 2007; Guarani et al. 2014; Huttlin et al. 2015
TIMMDC1	Complex I assembly factor TIMMDC1, mitochondrial	MCIA factor	Guarani et al. 2014; Huttlin et al. 2015
TMEM126B	Complex I assembly factor TMEM126B, mitochondrial	MCIA factor	Guarani et al. 2014; Huttlin et al. 2015
NDUFAF3	NADH dehydrogenase [ubiquinone] 1 alpha subcomplex assembly factor 3	CI assembly factor	Guarani et al. 2014
NDUFA12	NADH dehydrogenase [ubiquinone] 1 alpha subcomplex subunit 12	CI accessory subunit	Guarani et al. 2014
NDUFA13	NADH dehydrogenase [ubiquinone] 1 alpha subcomplex subunit 13	CI accessory subunit	Guarani et al. 2014
NDUFA8	NADH dehydrogenase [ubiquinone] 1 alpha subcomplex subunit 8	CI accessory subunit	Huttlin et al. 2015
NDUFA9	NADH dehydrogenase [ubiquinone] 1 alpha subcomplex subunit 9, mitochondrial	CI accessory subunit	Dunning et al. 2007
NDUFB6	NADH dehydrogenase [ubiquinone] 1 beta subcomplex subunit 6	CI accessory subunit	Dunning et al. 2007
NDUFB11	NADH dehydrogenase [ubiquinone] 1 beta subcomplex subunit 11, mitochondrial	CI accessory subunit	Guarani et al. 2014
NDUFS5	NADH dehydrogenase [ubiquinone] iron-sulfur protein 5	CI accessory subunit	Guarani et al. 2014
NDUFS1	NADH-ubiquinone oxidoreductase 75 kDa subunit, mitochondrial	CI core subunit	Guarani et al. 2014
NDUFS3	NADH dehydrogenase [ubiquinone] iron-sulfur protein 3, mitochondrial	CI core subunit	Guarani et al. 2014; Huttlin et al. 2015
NDUFS7	NADH dehydrogenase [ubiquinone] iron-sulfur protein 7, mitochondrial	CI core subunit	Dunning et al. 2007
MT-ND1	NADH-ubiquinone oxidoreductase chain 1	CI core subunit	Dunning et al. 2007
MRPL1	39S ribosomal protein L1, mitochondrial	mitochondrial translation	Guarani et al. 2014

Supplementary Table 2. ECSIT protein interacting ETC partners. *Interactions curated by Intact (Orchard, 2014) and Biogrid (Chatr-aryamontri et al., 2015)*

Interactor	Description	ETC function	References
ACAD9	Acyl-CoA dehydrogenase family member 9, mitochondrial	MCIA factor	Guarani et al. 2014; Huttlin et al. 2015
NDUFAF1	Complex I intermediate-associated protein 30, mitochondrial	MCIA factor	Vogel et al. 2007; Guarani et al. 2014; Huttlin et al. 2015
TIMMDC1	Complex I assembly factor TIMMDC1, mitochondrial	MCIA factor	Guarani et al. 2014; Huttlin et al. 2015
TMEM126B	Complex I assembly factor TMEM126B, mitochondrial	MCIA factor	Guarani et al. 2014; Huttlin et al. 2015
NDUFAF3	NADH dehydrogenase [ubiquinone] 1 alpha subcomplex assembly factor 3	CI assembly factor	Guarani et al. 2014
NDUFAF4	NADH dehydrogenase [ubiquinone] 1 alpha subcomplex assembly factor 4	CI assembly factor	Huttlin et al. 2015
COA1	cytochrome c oxidase assembly factor 1 homolog (S. cerevisiae)	CI & CIV assembly factor	Huttlin et al. 2015
NDUFA13	NADH dehydrogenase [ubiquinone] 1 alpha subcomplex subunit 13	CI accessory subunit	Huttlin et al. 2015
NDUFA3	NADH dehydrogenase [ubiquinone] 1 alpha subcomplex subunit 3	CI accessory subunit	Huttlin et al. 2015
NDUFA8	NADH dehydrogenase [ubiquinone] 1 alpha subcomplex subunit 8	CI accessory subunit	Huttlin et al. 2015
NDUFB1	NADH dehydrogenase [ubiquinone] 1 beta subcomplex subunit 1	CI accessory subunit	Huttlin et al. 2015
NDUFB11	NADH dehydrogenase [ubiquinone] 1 beta subcomplex subunit 11, mitochondrial	CI accessory subunit	Guarani et al. 2014
NDUFB5	NADH dehydrogenase [ubiquinone] 1 beta subcomplex subunit 5, mitochondrial	CI accessory subunit	Huttlin et al. 2015
NDUFB8	NADH dehydrogenase [ubiquinone] 1 beta subcomplex subunit 8, mitochondrial	CI accessory subunit	Huttlin et al. 2015
NDUFC2	NADH dehydrogenase [ubiquinone] 1 subunit C2, isoform 2	CI accessory subunit	Huttlin et al. 2015
NDUFS5	NADH dehydrogenase [ubiquinone] iron-sulfur protein 5	CI accessory subunit	Guarani et al. 2014
MT-ND1	NADH-ubiquinone oxidoreductase chain 1	CI core subunit	Huttlin et al. 2015
MT-ND4	NADH-ubiquinone oxidoreductase chain 4	CI core subunit	Guarani et al. 2014; Huttlin et al. 2015
NDUFS1	NADH-ubiquinone oxidoreductase 75 kDa subunit, mitochondrial	CI core subunit	Guarani et al. 2014

NDUFS2	NADH dehydrogenase [ubiquinone] iron-sulfur protein 2, mitochondrial	CI core subunit	Guarani et al. 2014; Huttlin et al. 2015
NDUFS3	NADH dehydrogenase [ubiquinone] iron-sulfur protein 3, mitochondrial	CI core subunit	Guarani et al. 2014; Huttlin et al. 2015
NDUFS8	NADH dehydrogenase [ubiquinone] iron-sulfur protein 8, mitochondrial	CI core subunit	Huttlin et al. 2015
NNT	NAD(P) transhydrogenase, mitochondrial	proton pump across the membrane	Huttlin et al. 2015
TMEM70	Transmembrane protein 70, mitochondrial	CV assembly factor	Guarani et al. 2014
MRPS17	mitochondrial ribosomal protein S17	mitochondrial translation	Guarani et al. 2014
MTIF2	mitochondrial translational initiation factor 2	mitochondrial translation	Huttlin et al. 2015

Supplementary Table 3. ACAD9 protein interacting ETC partners. *Interactions curated by Intact (Orchard, 2014) and Biogrid (Chatr-aryamontri et al., 2015)*

Interactor	Description	ETC Function	References
ECSIT	Evolutionarily conserved signaling intermediate in Toll pathway, mitochondrial	MCIA factor	Mick et al. 2012; Guarani et al. 2014
NDUFAF1	Complex I intermediate-associated protein 30, mitochondrial	MCIA factor	Guarani et al. 2014
TIMMDC1	Complex I assembly factor TIMMDC1, mitochondrial	MCIA factor	Guarani et al. 2014
TMEM126B	Complex I assembly factor TMEM126B, mitochondrial	MCIA factor	Guarani et al. 2014
FOXRED1	FAD-dependent oxidoreductase domain-containing protein 1	CI assembly factor	Mick et al. 2012
NDUFAF3	NADH dehydrogenase [ubiquinone] 1 alpha subcomplex assembly factor 3	CI assembly factor	Guarani et al. 2014
NDUFA13	NADH dehydrogenase [ubiquinone] 1 alpha subcomplex subunit 13	CI accessory subunit	Guarani et al. 2014
NDUFS5	NADH dehydrogenase [ubiquinone] iron-sulfur protein 5, mitochondrial	CI accessory subunit	Guarani et al. 2014
MT-ND6	NADH-ubiquinone oxidoreductase chain 6	CI core subunit	Guarani et al. 2014
NDUFS2	NADH dehydrogenase [ubiquinone] iron-sulfur protein 2, mitochondrial	CI core subunit	Guarani et al. 2014
NDUFS3	NADH dehydrogenase [ubiquinone] iron-sulfur protein 3, mitochondrial	CI core subunit	Guarani et al. 2014
NDUFS7	NADH dehydrogenase [ubiquinone] iron-sulfur protein 7, mitochondrial	CI core subunit	Guarani et al. 2014
AT5F1	ATP synthase F(0) complex subunit B1, mitochondrial	CV subunit	Mick et al. 2012
ATP5A1	ATP synthase subunit alpha, mitochondrial	CV subunit	Mick et al. 2012
ATP5B	ATP synthase subunit beta, mitochondrial	CV subunit	Mick et al. 2012
ATP5C1	ATP synthase subunit gamma, mitochondrial	CV subunit	Mick et al. 2012
ATP5D	ATP synthase subunit delta, mitochondrial	CV subunit	Mick et al. 2012
ATP5H	ATP synthase subunit d, mitochondrial	CV subunit	Mick et al. 2012
ATP5L	ATP synthase subunit g, mitochondrial	CV subunit	Mick et al. 2012
ATPO	ATP synthase subunit O, mitochondrial	CV subunit	Mick et al. 2012
MT-ATP6	ATP synthase subunit a, mitochondrial	CV subunit	Mick et al. 2012
ABCF2	ATP-binding cassette sub-family F member 2	ATP binding	Guarani et al. 2014

SIRT4	NAD-dependent protein lipoamidase sirtuin-4, mitochondrial	NAD+ binding	Mathias et al. 2014
ICT1	Peptidyl-tRNA hydrolase ICT1, mitochondrial	mitochondrial translation	Guarani et al. 2014
RPL26L1	60S ribosomal protein L26-like 1	mitochondrial translation	Guarani et al. 2014

Supplementary Table 4. TMEM126B protein interacting ETC partners. *Interactions curated by Intact (Orchard, 2014) and Biogrid (Chatr-aryamontri et al., 2015)*

Interactor	Description	ETC function	References
ECSIT	Evolutionarily conserved signaling intermediate in Toll pathway, mitochondrial	MCIA factor	Guarani et al. 2014
ACAD9	Acyl-CoA dehydrogenase family member 9, mitochondrial	MCIA factor	Guarani et al. 2014
NDUFAF1	Complex I intermediate-associated protein 30, mitochondrial	MCIA factor	Guarani et al. 2014
TIMMDC1	Complex I assembly factor TIMMDC1, mitochondrial	MCIA factor	Guarani et al. 2014
NDUFA13	NADH dehydrogenase [ubiquinone] 1 alpha subcomplex subunit 13	CI accessory subunit	Guarani et al. 2014

Supplementary Table 5. TIMMDC1 protein interacting ETC partners. *Interactions curated by Intact (Orchard, 2014) and Biogrid (Chatr-aryamontri et al., 2015)*

Interactor	Description	ETC Function	References
ACAD9	Acyl-CoA dehydrogenase family member 9, mitochondrial	MCIA factor	Guarani et al. 2014
ECSIT	Evolutionarily conserved signaling intermediate in Toll pathway, mitochondrial	MCIA factor	Guarani et al. 2014
NDUFAF1	Complex I intermediate-associated protein 30, mitochondrial	MCIA factor	Guarani et al. 2014
TMEM126B	Complex I assembly factor TMEM126B, mitochondrial	MCIA factor	Guarani et al. 2014
NDUFAF3	NADH dehydrogenase [ubiquinone] 1 alpha subcomplex assembly factor 3	CI assembly factor	Guarani et al. 2014
NDUFAF4	NADH dehydrogenase [ubiquinone] 1 alpha subcomplex assembly factor 4	CI assembly factor	Guarani et al. 2014
NDUFA12	NADH dehydrogenase [ubiquinone] 1 alpha subcomplex subunit 12	CI accessory subunit	Guarani et al. 2014
NDUFA13	NADH dehydrogenase [ubiquinone] 1 alpha subcomplex subunit 13	CI accessory subunit	Guarani et al. 2014
NDUFA3	NADH dehydrogenase [ubiquinone] 1 alpha subcomplex subunit 3	CI accessory subunit	Guarani et al. 2014
NDUFA8	NADH dehydrogenase [ubiquinone] 1 alpha subcomplex subunit 8	CI accessory subunit	Guarani et al. 2014
NDUFA9	NADH dehydrogenase [ubiquinone] 1 alpha subcomplex subunit 9, mitochondrial	CI accessory subunit	Guarani et al. 2014
NDUFB10	NADH dehydrogenase [ubiquinone] 1 beta subcomplex subunit 10, mitochondrial	CI accessory subunit	Guarani et al. 2014
NDUFB4	NADH dehydrogenase [ubiquinone] 1 beta subcomplex subunit 4, mitochondrial	CI accessory subunit	Guarani et al. 2014
NDUFB5	NADH dehydrogenase [ubiquinone] 1 beta subcomplex subunit 5, mitochondrial	CI accessory subunit	Guarani et al. 2014
NDUFB6	NADH dehydrogenase [ubiquinone] 1 beta subcomplex subunit 6, mitochondrial	CI accessory subunit	Guarani et al. 2014
NDUFB8	NADH dehydrogenase [ubiquinone] 1 beta subcomplex subunit 8, mitochondrial	CI accessory subunit	Guarani et al. 2014
NDUFB9	NADH dehydrogenase [ubiquinone] 1 beta subcomplex subunit 9, mitochondrial	CI accessory subunit	Guarani et al. 2014
NDUFS5	NADH dehydrogenase [ubiquinone] iron-sulfur protein 5, mitochondrial	CI accessory subunit	Guarani et al. 2014
NDUFV3	NADH dehydrogenase [ubiquinone] flavoprotein 3, mitochondrial	CI accessory subunit	Guarani et al. 2014

MT-ND1	NADH-ubiquinone oxidoreductase chain 1	CI core subunit	Guarani et al. 2014
MT-ND2	NADH-ubiquinone oxidoreductase chain 2	CI core subunit	Guarani et al. 2014
MT-ND4	NADH-ubiquinone oxidoreductase chain 4	CI core subunit	Guarani et al. 2014
NDUFS2	NADH dehydrogenase [ubiquinone] iron-sulfur protein 2, mitochondrial	CI core subunit	Guarani et al. 2014
NDUFS7	NADH dehydrogenase [ubiquinone] iron-sulfur protein 7, mitochondrial	CI core subunit	Guarani et al. 2014
NDUFS8	NADH dehydrogenase [ubiquinone] iron-sulfur protein 8, mitochondrial	CI core subunit	Guarani et al. 2014
NDUFV1	NADH dehydrogenase [ubiquinone] flavoprotein 1, mitochondrial	CI core subunit	Guarani et al. 2014
YME1L1	ATP-dependent zinc metalloprotease YME1L1	CI activity	Guarani et al. 2014
UQCR10	Cytochrome b-c1 complex subunit 9	CIII subunit	Guarani et al. 2014
COA3	Cytochrome c oxidase assembly factor 3 homolog, mitochondrial	CIV subunit	Mick et al. (2012)
COX5B	Cytochrome c oxidase subunit 5B, mitochondrial	CIV subunit	Guarani et al. 2014
COX6B1	Cytochrome c oxidase subunit 6b1	CIV subunit	Guarani et al. 2014
COX7A2L	Cytochrome c oxidase subunit 7A-related protein, mitochondrial	CIV subunit	Guarani et al. 2014
HCCS	Cytochrome c-type heme lyase	CIV subunit	Guarani et al. 2014
ATP5I	ATP synthase subunit g, mitochondrial	CV subunit	Mick et al. 2012
LRPPRC	Leucine-rich PPR motif-containing protein, mitochondrial	mitochondrial translation	Hein et al. (2015)
TIMM8A	Mitochondrial import inner membrane translocase subunit Tim8 A	mitochondrial transport	Guarani et al. 2014

SUPPLEMENTARY REFERENCES

Chatr-Aryamontri, A., Breitkreutz, B.J., Oughtred, R., Boucher, L., Heinicke, S., Chen, D.C., Stark, C., Breitkreutz, A., Kolas, N., O'donnell, L., Reguly, T., Nixon, J., Ramage, L., Winter, A., Sellam, A., Chang, C., Hirschman, J., Theesfeld, C., Rust, J., Livstone, M.S., Dolinski, K., and Tyers, M. (2015). The BioGRID interaction database: 2015 update. *Nucleic Acids Research* 43**,** D470-D478.

De Castro, E., Sigrist, C.J.A., Gattiker, A., Bulliard, V., Langendijk-Genevaux, P.S., Gasteiger, E., Bairoch, A., and Hulo, N. (2006). ScanProsite: detection of PROSITE signature matches and ProRule-associated functional and structural residues in proteins. *Nucleic Acids Research* 34**,** W362-W365.

Hall, T. (2011) BioEdit: An important software for molecular biology. *GERF Bulletin of Biosciences* 2(1), 60-61.

Larkin M.A., Blackshields, G., Brown, N.P., Chenna, R., McGettigan, P.A., McWilliam, H., Valentin, F., Wallace, I.M., Wilm, A., Lopez, R., Thompson, J.D., Gibson, T.J and Higgin,s D.G. (2007) Clustal W and Clustal X version 2.0 *Bioinformatics* 23(21), 2947-8.

Orchard, S. (2014). Data Standardization and Sharing The work of the HUPO-PSI. *Biochimica Et Biophysica Acta-Proteins and Proteomics* 1844**,** 82-87.
